# Complementary Medicine Linked to a Significant Prostate-Specific Antigen (PSA) Reduction in a 61-Year-Old Man: A Case Report

**DOI:** 10.7759/cureus.79510

**Published:** 2025-02-23

**Authors:** Elizabeth Huang, Jake Wong, Theresa Rohr-Kirchgraber

**Affiliations:** 1 General Practice, Augusta University/University of Georgia Medical Partnership, Athens, USA; 2 Internal Medicine, Medical College of Georgia, Augusta University, Athens, USA

**Keywords:** adt, complementary medicine, herbal medicine, prostate cancer, psa

## Abstract

Medical treatment for metastatic prostate cancer (mPC) involves androgen deprivation therapy (ADT) and selective use of surgery, chemotherapy, and/or radiation therapy. Our 61-year-old Chinese man with mPC had a prostate-specific antigen (PSA) drop of over 100 ng/mL with herbal medicine. Presenting atypically with a supraclavicular mass and an initial PSA greater than 20 times the upper normal limit, he self-medicated with herbs while pending insurance approval of prescribed ADT. After two months, his PSA dropped 10-fold. Subsequent chemotherapy dropped his PSA to within the normal range. This highlights the potential of traditional herbal medicine to benefit patients and provide complementary therapy to established drug regimens.

## Introduction

Metastatic prostate cancer (mPC), mostly in men over 65 years old, can present with a variety of symptoms depending on the organ affected. Symptoms may include fatigue, weight loss, sexual dysfunction, urinary problems, lymphedema, and bone pain. Asian men in the U.S. have a relatively low prevalence (age-adjusted values of 2 to 10 per 100,000 men) [1].

Because a prostate-specific antigen (PSA) may be transiently elevated by prostatitis, recent sexual activity, and other conditions, the American Urological Association no longer recommends a single PSA cutoff for biopsy. They suggest including factors such as age, ethnicity, comorbidities, prior medical history, and a repeat PSA [2]. Our patient with mPC presented without classic symptoms and had an atypical path to diagnosis starting with lymph node biopsy.

Treatment of localized prostate cancer usually involves androgen deprivation therapy (ADT) and/or radical prostatectomy, while treatment of mPC typically involves the use of ADT, chemotherapy, and/or radiopharmaceuticals [2]. The choice of therapy depends on the stage of cancer, as determined by a combination of PSA level at diagnosis, histological grade, number of lymph nodes affected, and metastasis to distant sites [2].

In traditional medicine originating from Asia and the Middle East, various herbs are believed to have anti-inflammatory and antioxidant properties. Used in treating hypertension, arthritis, and various cancers, many are not well studied experimentally and have not shown significant efficacy for prostate cancer [3]. Our patient turned to traditional herbal medicine while awaiting approval for the prescribed medical therapy and had an interesting result.

This article was previously presented as an abstract at the Society for General Internal Medicine (SGIM) Annual Meeting on May 17, 2024.

## Case presentation

The wife of a 61-year-old Chinese man noted an enlarging left-sided neck mass in February 2021, and the patient was evaluated within two weeks. He had a history of nephrolithiasis, treated latent tuberculosis, and ongoing dental implants. His father had localized prostate and metastatic bladder cancer. His mother had end-stage renal disease and stomach cancer. The patient had no history of tobacco, alcohol, or recreational drug use and worked as a radiology and nuclear medicine technician for over 20 years. Except for a non-mobile, non-tender 2 cm supraclavicular mass, the physical exam and vital signs were unremarkable. His first-ever PSA was 87 ng/mL. Urinalysis showed trace blood, while the complete metabolic panel (CMP), complete blood count (CBC), and thyroid-stimulating hormone (TSH) were within normal limits (WNL). The hepatitis C antibody was non-reactive (Table 1). One week later, the lymph node biopsy was taken, revealing prostate-specific antigen-positive (PSA+) and prostatic acid phosphatase-positive (PSAP+) non-small cell carcinoma, most consistent with prostatic origin with no definitive background lymph node tissue (digital pathology image is not available, per conducting laboratory). The prostate was not biopsied.

**Table 1:** Lab values pre- and post-herbal therapy (pre-chemotherapy) POC: point of care; BUN: blood urea nitrogen; ALP: alkaline phosphatase; AST: aspartate aminotransferase; ALT: alanine aminotransferase; GFR: glomerular filtration rate; WBC: white blood cell count; RBC: red blood cell count; Hgb: hemoglobin; Hct: hematocrit; MCV: mean corpuscular volume; MCH: mean corpuscular hemoglobin; MCHC: mean corpuscular hemoglobin concentration; RDW: red cell distribution width; Plt: platelet count; MPV: mean platelet volume; TSH: thyroid-stimulating hormone; PSA: prostate-specific antigen

	Measurement	Initial Pre-herbal Therapy Lab Results at PCP (February 2, 2021)	Pre-herbal Therapy Lab Results at Oncologist (March 9, 2021)	Post-herbal Therapy, Pre-chemotherapy Lab Results (April 14, 2021)	Post-chemotherapy Lab Results (August 2, 2021)	Reference Values
POC Urinalysis Dipstick						
	Color/appearance	Yellow/clear	-	-	-	-
	Clarity	Clear	-	-	-	-
	Glucose	Negative	-	-	-	Negative
	Bilirubin	Negative	-	-	-	Negative-Large
	Ketones	Negative	-	-	-	Negative-160 (large)
	Specific gravity	1.015	-	-	-	1.000-1.030
	Blood	Trace-lysed	-	-	-	Negative-Large (3+)
	pH	7.0	-	-	-	5.0-8.5
	Protein	Negative	-	-	-	Negative - ≥2000 (4+)
	Urobilinogen	0.2	-	-	-	0.2-8.0
	Nitrite	Negative	-	-	-	Negative
	Leukocytes	Negative	-	-	-	Negative
Comprehensive Metabolic Panel						
	Sodium	141 mmol/L	139 mmol/L	141 mmol/L	138 mmol/L	136-145 mmol/L
	Potassium	4.5 mmol/L	4.7 mmol/L	4.3 mmol/L	4.2 mmol/L	3.5-5.1 mmol/L
	Chloride	103 mmol/L	102 mmol/L	104 mmol/L	106 mmol/L	98-107 mmol/L
	CO2	28 mmol/L	29 mmol/L	26 mmol/L	27 mmol/L	22-29 mmol/L
	Glucose	80 mg/dL	99 mg/dL	100 mg/dL	104 mg/dL	70-99 mg/dL
	BUN	12 mg/dL	12 mg/dL	18 mg/dL	16 mg/dL	8-23 mg/dL
	Creatinine, serum	0.74 mg/dL	0.86 mg/dL	0.76 mg/dL	0.68 mg/dL	0.7-1.2 mg/dL
	Protein, total	7.4 g/dL	6.6 g/dL	6.6 g/dL	5.7 g/dL	6.4-8.4 g/dL
	Albumin	4.3 g/dL	4.2 g/dL	4.1 g/dL	3.7 g/dL	3.5-5.2 g/dL
	Calcium	9.1 mg/dL	9.4 mg/dL	9.0 mg/dL	8.4 mg/dL	8.8-10.2 mg/dL
	Bilirubin, total	0.9 mg/dL	0.5 mg/dL	0.5 mg/dL	0.3 mg/dL	0.0-1.2 mg/dL
	ALP	78 IU/L	67 IU/L	86 IU/L	113 IU/L	40-129 IU/L
	AST	21 IU/L	18 IU/L	22 IU/L	16 IU/L	0-40 IU/L
	ALT	12 IU/L	17 IU/L	18 IU/L	14 IU/L	0-41 IU/L
	Globulin	3.1 g/dL	2.4 g/dL	2.5 g/dL	2.0 IU/L	2.4-4.0 g/dL
	Anion gap	15		11	-	12-20
	GFR	>90 mL/min/1.73 m^2^	>90 mL/min/1.73 m^2^	>90 mL/min/1.73 m^2^	>90 mL/min/1.73 m^2^	>59 mL/min/1.73 m^2^
CBC With Differential						
	WBC	5.21 cells/µL	5.5 cells/µL	5.4 cells/µL	11.7 cells/µL	3.5-10.5 cells/µL
	RBC	4.73 cells/µL	4.55 cells/µL	4.37 cells/µL	3.04 cells/µL	4.32-5.72 cells/µL
	Hgb	14.8 g/dL	14.0 g/dL	13.6 g/dL	10.0 g/dL	13.5-17.5 g/dL
	Hct	47.4%	42.5%	40.3%	30.0%	39-50%
	MCV	100.2 fL	93.4 fL	92.2 fL	98.7 fL	81.0-95.0 fL
	MCH	31.3 pg	30.8 pg	31.1 pg	32.9 pg	36.0-34.0 pg
	MCHC	31.2 g/dL	32.9 g/dL	33.7 g/dL	33.3 g/dL	32.0-36.0 g/dL
	RDW	13.2%	12.6%	12.3%	14.8%	11.8-15.6%
	Plt	220 cells/µL	201 cells/µL	172 cells/µL	150 cells/µL	150-450 cells/µL
	MPV	11.0 fL	9.4 fL	10.5 fL	10.0 fL	9.4-12.4 fL
	% Immature granulocytes	0%	0.20%	0.0%	1.50%	-
	% Neutrophils	43.5%	42.2%	41.6%	74%	-
	% Lymphocytes	43.6%	42.1%	40.1%	13.1%	-
	% Monocytes	9.4%	10.8%	9.6%	9.6%	-
	% Eosinophils	2.7%	3.8%	7.6%	1.3%	-
	% Basophils	0.8%	0.9%	1.1%	0.5%	-
	Absolute immature granulocytes	0	0.01 cells/µL	0	0.18 cells/µL	0-0.10 cells/µL
	Absolute neutrophils	2.27 cells/µL	2.3 cells/µL	2.3 cells/µL	8.7 cells/µL	1.7-7.0 cells/µL
	Absolute lymphocytes	2.26 cells/µL	2.3 cells/µL	2.2 cells/µL	1.5 cells/µL	1.5-4.0 cells/µL
	Absolute monocytes	0.49 cells/µL	0.60 cells/µL	0.52 cells/µL	1.13 cells/µL	0.3-0.9 cells/µL
	Absolute eosinophils	0.14 cells/µL	0.2 cells/µL	0.4 cells/µL	0.2 cells/µL	0.1-0.5 cells/µL
	Absolute basophils	0.04 cells/µL	0.1 cells/µL	0.1 cells/µL	0.1 cells/µL	0.0-0.3 cells/µL
TSH	1.43 uIU/mL		-	-	-	0.27-4.20 uIU/mL
PSA	86.6 ng/mL		135 ng/mL	9.9 ng/mL	0.5 ng/mL	0.0-4.0 ng/mL
Hepatitis C Antibody	Non-reactive		-	-	-	Non-reactive

**Figure 1:**
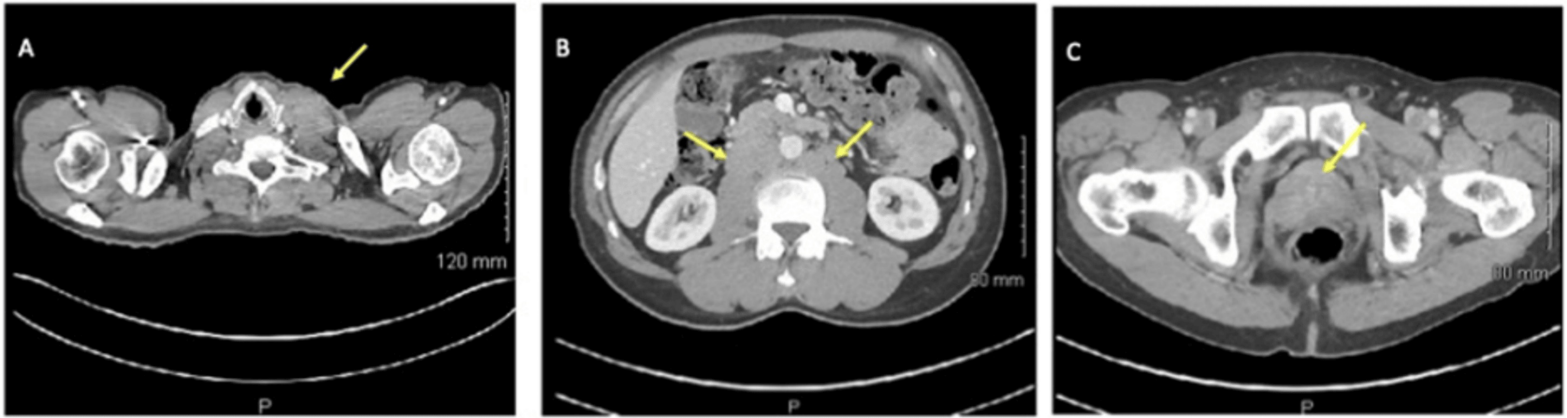
CT with IV contrast images (A) Transverse view at the neck of left supra-clavicular lymphadenopathy. (B) Transverse view at the upper abdomen of para-aortic lymphadenopathy. (C) Transverse view at the pelvis of hyper-intensity in the right peripheral zone of the prostate suspicious of malignancy.

One month later at his initial oncology visit, his PSA was 135 ng/mL. Repeat CMP and CBC were WNL (Table 1) and no other blood or urine tests were taken at this visit. Whole-body bone imaging revealed no evidence of osteoblastic lesions. A CT scan of the chest, abdomen, and pelvis with IV contrast (Figure 1) revealed enlarged left supraclavicular lymph nodes (1.7 cm x 2.2 cm) with no intrathoracic or axillary adenopathy. Retroperitoneal adenopathy was noted (index lymph nodes: left periaortic chain (1.9 cm x 3.0 cm), retrocaval chain (2.0 cm x 3.7 cm), and aortocaval chain (2.2 cm x 4.1 cm)). Pelvic adenopathy was also present (index lymph nodes: right common iliac chain (0.9 cm x 1.0 cm), left common iliac bifurcation (2.0 cm x 1.8 cm)). Additionally, hyperdensity was observed involving the right peripheral zone of the prostate (likely reflecting the primary prostate malignancy), and no aggressive osseous lesions were identified. Diagnosed with stage IV prostate cancer, the patient agreed to start treatment with leuprolide acetate the following week, and then begin docetaxel followed by abiraterone after finishing his dental implant procedures. Lymph node biopsy and CT were sufficient for diagnosis and staging; the prostate was never biopsied during the course of this entire investigation, per National Comprehensive Cancer Network (NCCN) guidelines for evaluating mPC with very elevated PSA.

Unfortunately, prior authorization delayed the treatment timeline. Fearing that he would receive therapy too late in his disease course, he began drinking smoothies three times a day consisting of herbal ingredients. The recipe for three servings was as follows: 50 g sabah snake grass (*Clinacanthus nutans*), 50 g cat’s whiskers (*Orthosiphon stamineus*), one large ginger root, 80 g Manuka honey, five tablespoon flaxseed, six bunches of dandelion leaves with roots, and pH 11.5 alkaline water were blended together in a commercial blender. The patient then drank one serving with each meal daily for the next month. All plants were grown organically in the patient's basement, and he did not notice any side effects from these supplements.

**Figure 2:**
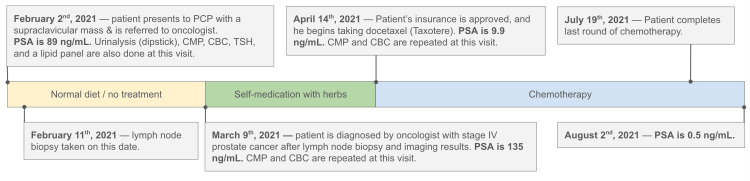
Timeline of clinical course CMP: complete metabolic panel; CBC: complete blood count; TSH: thyroid stimulating hormone; PSA: prostate-specific antigen; PCP: primary care physician

Five weeks later in April, insurance approved his therapy treatment, and the PSA, prior to ADT, had dropped to 9.9 ng/mL (Figure 2). The patient proceeded straight to docetaxel infusion therapy every three weeks with pre-cycle dexamethasone and continued his previous regimen of herbal smoothies. He declined abiraterone follow-up therapy and completed five out of six cycles of docetaxel, before stopping therapy in July due to neuropathy three months later. He was started on leuprolide acetate maintenance therapy. His final PSA in August was 0.5 ng/mL.

Since the completion of chemotherapy in 2021, the patient's PSA levels and overall status remained stable until PSA trended up to 3.7 ng/mL in January 2023. The patient started apalutamide, and PSA decreased to 0.6 ng/mL. Due to adverse effects of abdominal bloating and rash interfering with ambulation, he stopped apalutamide in March 2023. He declined concurrent abiraterone with prednisone therapy due to high co-pays, while continuing Lupron. PSA trended up to 3.9 ng/mL in August 2023. The patient received Provenge in August 2023, but PSA continued trending up to 7.8 ng/mL. In December 2023, the patient consented to abiraterone due to rising PSA, and PSA dropped to 0.04 ng/mL by March 2024. The patient was then switched to darolutamide in October 2024. As of the winter of 2025, the patient has remained stable, continuing darolutamide therapy, with repeat PSA labs every three months which have stayed below 1 ng/mL.

## Discussion

Metastasis to cervical or supraclavicular lymph nodes as an initial presenting symptom of mPC is rare. The incidence of supraclavicular lymphadenopathy (SCLN) was 0.22% among the Chinese patient population and <0.1% in a study conducted at Mayo Clinic in 1950-1968 [4]. The path of prostatic metastasis to cervical and supraclavicular lymph nodes is not certain, though a popular proposal is that mPC advances lymphatically via a stepwise fashion towards the SCLN [4], which may be the case for this patient. The CT scan showed the possible path of metastatic spread from the right peripheral zone of the prostate to the pelvic lymphatic chain, the common iliac lymphatic chains, the retroperitoneal lymphatic chains, and finally to the supraclavicular lymph nodes.

The first-line treatment of this metastatic castration-sensitive prostate cancer (mCSPC) is ADT with treatment intensification [2]. This patient, who was not able to receive ADT therapy (leuprolide) before docetaxel due to his insurance delay, used complementary medicine while awaiting approval.

*O. stamineus* is widely used in East and Southeast Asia as food medicine for a multitude of conditions from cancers to hypertension [3]. There has been a recent movement to research creating extracts with cytotoxic effects against prostate cancer cells. Certain extracts have been found to have significant in vitro antiproliferative effects against human DU-145 prostate cancer cells and in vitro cytotoxic effects against human PC3 prostate cancer cells. Notably, they had no effect on non-neoplastic cells [5].

*C. nutans*, an East/Southeastern Asian traditional herb, is used for a variety of non-neoplastic conditions, but there are no reports of its effects on prostate cancer cells. There is support for cytotoxic effects on other cancer cell types and it is proposed that if there is any impact on prostate cancer cells, it may be via an indirect immunomodulatory effect after phytochemical transformation by microbiota or human metabolism [5].

With 320 different types of honey in the world with different ratios and make-up of sugars and compounds [6], there is interest in investigating its possible preventative, chemotherapeutic, and antiproliferative effects. Although there are few well-controlled clinical trials on honey’s anti-neoplastic (particularly prostate cancer) effects, there is evidence that certain types of honey (such as manuka and thyme) have chemotherapeutic effects for radiation-induced mucositis of head and neck cancers [7]. Thyme honey may have prostate cancer prevention-related processes and acacia honey may contribute to antiproliferative activity, induction of apoptosis, anti-inflammatory effects, immunomodulatory activity, p53 regulation, and cell cycle arrest effects on PC3 prostate cancer cells [6].

Consuming flaxseed-derived enterolactone was found to be inversely associated with mPC proliferation in a presurgical clinical trial [8], and dandelion extract was found to induce cytotoxicity in combination with other compounds in breast cancer cells. As with natural honey, dandelion extracts have been credited with immunoprotective, antioxidant, and anticancer effects [9]. There is insufficient evidence that oral consumption of either flaxseed or dandelion extract is preventive for prostate cancer.

However, did this patient’s elevated PSA truly reflect prostate cancer? An elevated PSA can result from multiple etiologies, such as prostate cancer, prostatitis, artificial elevation via supplements (e.g., biotin), or a lab error. This patient did not report any urinary symptoms, did not take other medications or supplements, and had an elevated PSA that was confirmed by two separate physicians at two different labs. It should be noted that although the second PSA was taken four weeks after the lymph node biopsy, residual inflammation from the biopsy may still have contributed to the rise in PSA from 89 to 135. A possible mechanism for the decreased PSA two months after diagnosis is that the patient may have had silent prostatitis or prostate inflammation from the carcinoma which was treated by the possible anti-inflammatory effects of the herbs. Whether via anti-inflammatory, immunomodulatory, or anti-carcinogenic effects, his self-medication was likely contributory to his drop in PSA.

Complementary and alternative medicine (CAM) daunts many physicians due to its unfamiliar and unregulated territory, but cancer patients have reported increasing use of multiple forms of CAM. In surveys taken by CaPSURE (Cancer of the Prostate Strategic Urologic Research Endeavor) patients from 1996 to 2016, just over half of patients reported use of CAM, with an uptrend from 24% to 54% over these years [10]. In the U.S., research into CAMs may be conducted and supported by federal organizations such as the National Center for Complementary and Integrative Health (NCCIH). Knowing that several existing chemotherapeutic drugs have been derived from plants (e.g., vinca alkaloids, psoralen), further research into herbal remedies represents a promising avenue of isolating active ingredients that may one day be prescribed for mPC. However, a major concern with CAM is the unregulated nature of the products and what may be included in them. Although we did not find explicit examples of this in our case presentation, it is important to note that the CAM products used may still contain as-of-yet unknown compounds that contributed to the effects seen.

Patient perspective

The following is the perspective of the patient: "I turned to herbal medicine because after my neck swelled, it was hard to eat. I went to my primary care physician (PCP), and my PSA was 87. Then I went to a gastroenterologist for help with my eating problem. The doctor said he could not help me because this was due to my prostate cancer, which only the oncologist could help with. I went to the oncologist, who said I'd have to wait for maybe more than a month to get approved for chemotherapy. So I used the herbs my father used, which seemed to help improve my father's bladder cancer. I used herbal smoothies, heating pads, TDP lamps, and original point treatment (whole body points massages with ginger) for around one month, to help me eat. I thought that even if it did not work, it would not hurt. I could even 'over-dose', and it would be fine, because they are vegetables too. And I knew there would be fewer side effects than if I took Western medicine. After this, my PSA reduced to less than 10 before I started chemotherapy."

## Conclusions

This case describes a rare initial presentation of mPC. Left SCLN (Virchow’s node) is often interpreted as a sign of abdominopelvic cancers, most commonly gastric, pancreatic, gallbladder and hepatobiliary, esophageal, testicular, and ovarian. However, it is an uncommon presenting sign of mPC, which prefers to spread retroperitoneally to bone. This case thus reminds physicians to maintain a broad differential and consider alternate diagnoses that may exist without their classic clinical features.

Additionally, this case highlights the potential of herbal medicine when used as a complementary therapy. Although it is uncertain if there was concurrent prostatitis during the initial PSA measurement, it is possible that the patient's herbal smoothies contributed to the greater than 100 ng/mL decrease in PSA levels. It is important to remember that this case report is of only one patient; CAM still should be pursued cautiously due to FDA regulations. Nevertheless, this case reminds physicians to partner with their patients and critically evaluate possible adjunct treatments for patients with difficulty accessing care.
